# Incidence and management of secondary deformities after megaendoprosthetic proximal femur replacement in skeletally immature bone sarcoma patients

**DOI:** 10.1007/s00402-024-05334-1

**Published:** 2024-05-03

**Authors:** Wiebke K. Guder, Nina M. Engel, Arne Streitbürger, Christina Polan, Marcel Dudda, Lars E. Podleska, Markus Nottrott, Jendrik Hardes

**Affiliations:** 1 Department of Orthopedic Oncology, University Hospital Essen, Hufelandstrasse 55, Essen, 45147 Germany; 2 Department of Trauma, Hand and Reconstructive Surgery, University Hospital Essen, Hufelandstrasse 55, Essen, 45147 Germany

**Keywords:** Proximal femur replacement, Megaendoprosthesis, Hip dysplasia, Genu valgum, Pediatric, Skeletal immaturity

## Abstract

**Introduction:**

Megaendoprosthetic reconstruction of bone defects in skeletally immature patients has led to the development of unique complications and secondary deformities not observed in adult patient cohorts. With an increasing number of megaendoprosthetic replacements performed, orthopedic oncologists still gain experience in the incidence and type of secondary deformities caused. In this study, we report the incidence, probable cause and management outcome of two secondary deformities after megaendoprosthetic reconstruction of the proximal femur: hip dysplasia and genu valgum.

**Materials and methods:**

Retrospective analysis of 14 patients who underwent primary and/or repeat reconstruction/surgery with a megaendoprosthetic proximal femur replacement between 2018 and 2022.

**Results:**

Mean patient age was 9.1 years (range 4–17 years). Stress shielding was observed in 71.4%. Hip dislocation was the most frequent complication (50%). While four dislocations occurred without an underlying deformity, secondary hip dysplasia was identified in 58.3% (*n* = 7/12) of intraarticular resections and reconstructions, leading to dislocation in 71.4% (*n* = 5/7). A genu valgum deformity was observed in 41.6% (*n* = 5/12). The incidence of secondary hip dysplasia and concomitant genu valgum was 42.9% (*n* = 3/7). Triple pelvic osteotomy led to rebound hip dysplasia in two cases (patients aged < 10 years), whereas acetabular socket replacement led to stable hip joints over the course of follow-up. Temporary hemiepiphyseodesis was applied to address secondary genu valgum.

**Conclusions:**

Patients aged < 10 years were prone to develop secondary hip dysplasia and genu valgum following proximal femur replacement in this study. Management of secondary deformities should depend on remaining skeletal growth. Stress shielding was observed in almost all skeletally immature patients.

**Supplementary Information:**

The online version contains supplementary material available at 10.1007/s00402-024-05334-1.

## Introduction

Megaendoprostheses in skeletally immature bone sarcoma patients are superseding other surgical treatment options such as amputation, rotation plasty and biological reconstructions over the last few decades [1]. Despite the need for repeat surgeries and an increased risk for implant-associated complications, patients and parents are more amenable to reconstructions using expandable growing prostheses, which are associated with both a satisfactory emotional acceptance and functional results [1–6]. Outcomes of these patients reconstructed with endoprostheses are monitored closely for complications associated with the growing skeleton (and their best course of treatment), which have not been observed in existing adult patient collectives in the past [1–3; 7–9]. So far, most of the existing studies focusing on pediatric patients report on outcomes and complications of endoprosthetic reconstructions centered around the knee joint, which is the most frequent tumor site [1–3; 7–9]. In this regard, the term “pediatric” failure has been coined to describe the number of repeat surgeries needed to achieve equal leg length and complete lengthening potential of the endoprosthetic reconstruction [1]. However, aside from these pediatric failures, complications affecting the growing skeleton surrounding the endoprosthetic reconstruction have been observed as well. A great variability in physis growth adjacent to the reconstruction (i.e. affecting the proximal tibia growth plate after distal femur replacement) has been noted [7–9]. Most frequently, growth inhibition following passive implant insertion of sliding stems crossing the affected physis were reported [8, 9]. The only significant contributor to the development of a growth disturbance observed, thus far, was younger patient age in a study by Shehadeh et al. [9].

Due to a lower incidence of bone sarcoma of the proximal femur, endoprosthetic reconstructions using standard or expandable prostheses in skeletally immature patients in this location are indicated less frequently than around the knee [10]. This is reflected in the still smaller number of studies and included patients reporting on outcomes and complications associated with proximal femur replacements in a pediatric collective [11, 12]. The most frequent complications reported for this site were hip dislocation and instability [12]. A case of iatrogenic physeal growth arrest of the distal femur was also observed after proximal femur replacement [13].

In this study, we report the incidence and probable cause of observed secondary deformities and complications, as well as their management and outcome in 14 skeletally immature patients who underwent endoprosthetic proximal femur replacement. Based on these findings, we present our current standard of care, which is discussed against corresponding findings in literature.

## Materials and methods

Fourteen skeletally immature patients (aged < 18 years) who underwent primary malignant bone tumor resection and reconstruction of the proximal femur or revision procedures at this tertiary Orthopedic Oncology department between 2018 and 2022 were identified from a surgical database. Patient data was prospectively collected from patient files in the hospital information system.

### Patient characteristics

Patient age at the time of primary tumor resection and proximal femur replacement was a mean of 9.1 years (range 4–17 years). In all patients, an incisional biopsy was performed. Histopathological analysis confirmed Ewing sarcoma in eight and osteosarcoma in four patients. Hemangioendothelioma and bone metastasis of osteosarcoma (#8) were diagnosed in one case each. All but one patients (#11 - hemangioendothelioma) completed neoadjuvant chemotherapy prior to the tumor resection and proximal femur reconstruction. Femoral growth plates at the time of primary tumor resection were judged to be open in ten (aged ≤ 10 years), intermediate in two (aged 11 and 13 years) and closed in two (aged 16 and 17) patients. Intraarticular resections were performed in twelve and extraarticular resections in two patients (who presented with an intraarticular pathological fracture). The mean resection length was 176 mm (range 80–320 mm). Patient #5 underwent rotation plasty of the contralateral right leg for the primary tumor and megaendoprosthetic reconstruction of the left proximal femur for a solitary, synchronous bone metastasis. A more detailed overview on patient characteristics is given in Table 1.

**Table 1:** Patient characteristics, implant properties and incidence of secondary deformities

#	Age (yr)	Diagnosis	Resection	Resection length (mm)	Reconstruction	Hip socket (mm)	Bipolar head (mm)	Femoral head (mm)	Distal stem	Stem length (mm)	Hip dysplasia	Hip dislocation	Genu valgum	LLD (mm)	FU (mo)
1	8	Ewing	intra	210	Xpand small	-	38	-	CM hollow	80	1	1	-	15	AWD 55
2	11	Ewing	intra	150	Standard	-	42	-	Xpand	90	-	-	(1)	40	NED 51
3	5	Ewing	intra	140	CM small	-	-	28	CM polished	70	1	1	(1)	0	NED 123
4	13	Ewing	intra	170	Standard	-	42	-	Standard	120	-	-	-	20	NED 43
5	10	Osteo	intra	320	Xpand	-	38	-	CM hollow plated	25	-	-	(1)	20	NED 42
6	8	Ewing	intra	140	Standard	-	38	-	CM polished	120	1	1	-	0	NED 112
7	7	Ewing	intra	270	Standard	-	38	-	CM hollow	55	1	-	1	28	NED 43
8	5	Osteo	intra	80	CM small	-	-	36	Standard humerus	75	1	1	-	0	NED 42
9	4	Ewing	intra	210	Xpand small	-	-	36	CM hollow plated	21	1	-	-	10	DOD (48)
10	4	Ewing	intra	160	Xpand small	-	-	36	CM hollow plated	45	1	1	1	NA	NED 64
11	17	HAE	intra	120	Standard	-	46	-	Xpand	90	-	-	-	0	AWD 64
12	16	Osteo	intra	180	Standard	-	46	-	Standard	120	-	-	-	0	NED 21
13	10	Osteo	extra	160	Xpand small	46	-	-	Standard humerus	75	-	1	-	10	NED 28
14	10	Osteo	extra	155	Xpand small	42	-	-	Standard humerus	75	-	1	-	0	NED 16

*Abbreviations* #: number; (yr): years; Ewing: Ewing sarcoma; Osteo: osteosarcoma; HAE: hemangioendothelioma; intra: intraarticular; extra: extraarticular; Xpand: MUTARS (implantcast®) growth prosthesis; CM: custom-made; 1: yes; LLD: leg length discrepancy; FU: follow-up; (mo): months

### Surgical technique and rehabilitation

Skeletally mature patients are operated on in a sideways position when undergoing proximal femur resection and reconstruction regardless of intra- or extraarticular resection at this department. However, a supine position in skeletally immature patients undergoing an intraarticular resection may also be used. Depending on the site of the largest soft tissue mass of the tumor, preparation and mobilization of the resection specimen is facilitated by an early femoral osteotomy or arthrotomy of the hip joint capsule. In extraarticular resection, acetabular osteotomy is the last surgical step before the resection specimen is removed. During reconstruction, attachment tubes are used for the refixation of muscles, tendons and the remaining joint capsule depending on defect size and intraoperative stability of the reconstruction (tendency for dislocation). Rehabilitation recommendations include partial weight bearing with 20 kg for six weeks after the operation (cementless stem fixation), followed by an incremental increase of weight bearing of 10 kg per week. For a period of six (later: twelve) weeks, flexion of the hip joint is restricted to 60° and adduction of 0°. Forceful internal or external rotation are avoided during that period as well.

In this patient cohort, eight patients were operated on in a supine (intraarticular resections: patients aged 5–11 years) and six patients in a sideways position (intraarticular resection: patients aged 10–17 years; extraarticular resection: patients aged 10 years) using a lateral approach. An attachment tube was used in eight patients (intraarticular *n* = 6; extraarticular *n* = 2).

### Implant properties

Megaendoprosthetic implants used in the analyzed patient cohort were manufactured by implantcast GmbH (Buxtehude, Germany). Whenever feasible with regard to patient proportions and soft tissue development, off-the-shelf implants of the MUTARS® system were used. Custom-made (CM) implants and growth megaendoprostheses (Xpand®) were used depending on the degree of skeletal immaturity. Standard or custom-made polished (sliding) or cementless stems were used depending on remaining femoral bone stock. More information on implant and stem types will be given in the following paragraph and Table 1.

#### Standard

off-the-shelf implants of the MUTARS® system and cementless, curved *standard femur stems* (120 mm length) (M10 screws).

#### Xpand

custom-made growth prosthesis implants and cementless, curved *Xpand® stems* (90 mm length) with standard dimensions (M10 screws).

#### Xpand small

custom-made growth prosthesis implants and cementless, straight *standard humerus stems* (75 mm length) with humerus dimensions (M8 screws).

#### CM small

custom-made non growth-prosthesis implants with smaller than standard dimensions (M8 screws).

#### CM hollow stem (non-)plated

custom-made hollow stems of varying lengths depending on remaining bone stock.

#### CM polished stem

custom-made polished stems of varying lengths to prevent ingrowth and bone loss in future revisions.

### Follow-up and management of secondary deformities

Patients were recommended to follow up in our outpatient clinic quarterly for the first two years, biannually until completion of the fifth and annually until completion of the tenth year after the operation. Follow-up appointments included patient history, clinical examination and radiographs in two planes of the reconstruction. Full-length standing anteroposterior (ap) and/or lateral view x-rays of the lower limb were added to examine limb alignment in the frontal and sagittal plane as needed. Limb salvage, complications according to Henderson et al. [14], as well as incidence, management and ouctcome of secondary deformities were documented in the hospital information system.

## Results

Limb salvage was 100% in this patient cohort of skeletally immature patients who underwent proximal femur replacement at a mean follow-up of 53.7 months (range 21–123 months). Eleven patients showed no evidence of disease at the latest follow-up, while two patients were alive with disease. One patient died of disease at 48 months after the operation.

### Complications according to Henderson

Eighteen revision operations (mean 1.3; range 0–4), including one open and three closed reductions of hip dislocations were performed during follow-up.

Mechanical type 1a failure (soft tissue failure) occurred nine times (*n* = 9/18; 50%) in seven patients (*n* = 7/14; 50%) who presented with hip dislocation. However, only four dislocations (*n* = 4/18; 22.2%; #5 *n* = 2; #13, #14) occurred early without an underlying secondary deformity zero (*n* = 1), one (*n* = 2) and five months (*n* = 1) after the primary operation. Another hip dislocation without underlying deformity occurred fourteen months after primary extraarticular resection and reconstruction in a patient (#13) who performed a maximal flexion, adduction and internal rotation of the hip joint. In two patients (*n* = 2/14; 14.3%; #13 and 14) extraarticular proximal femur resections were performed. Both were reconstructed using an attachment tube.

One patient presented with a torn power cord, connecting the receiver and motor of a growth prosthesis and secondary, symptomatic dislocation of the receiver unit (type 3a failure), while another patient suffered from a periprosthetic fracture (type 3b failure). Mechanical type 1b (aseptic wound dehiscence), type 2 (aseptic loosening) and non-mechanical complications such as periprosthetic infection (type 4) or local recurrence (type 5) did not occur in this patient cohort.

### Stress shielding

**Fig. 1:**
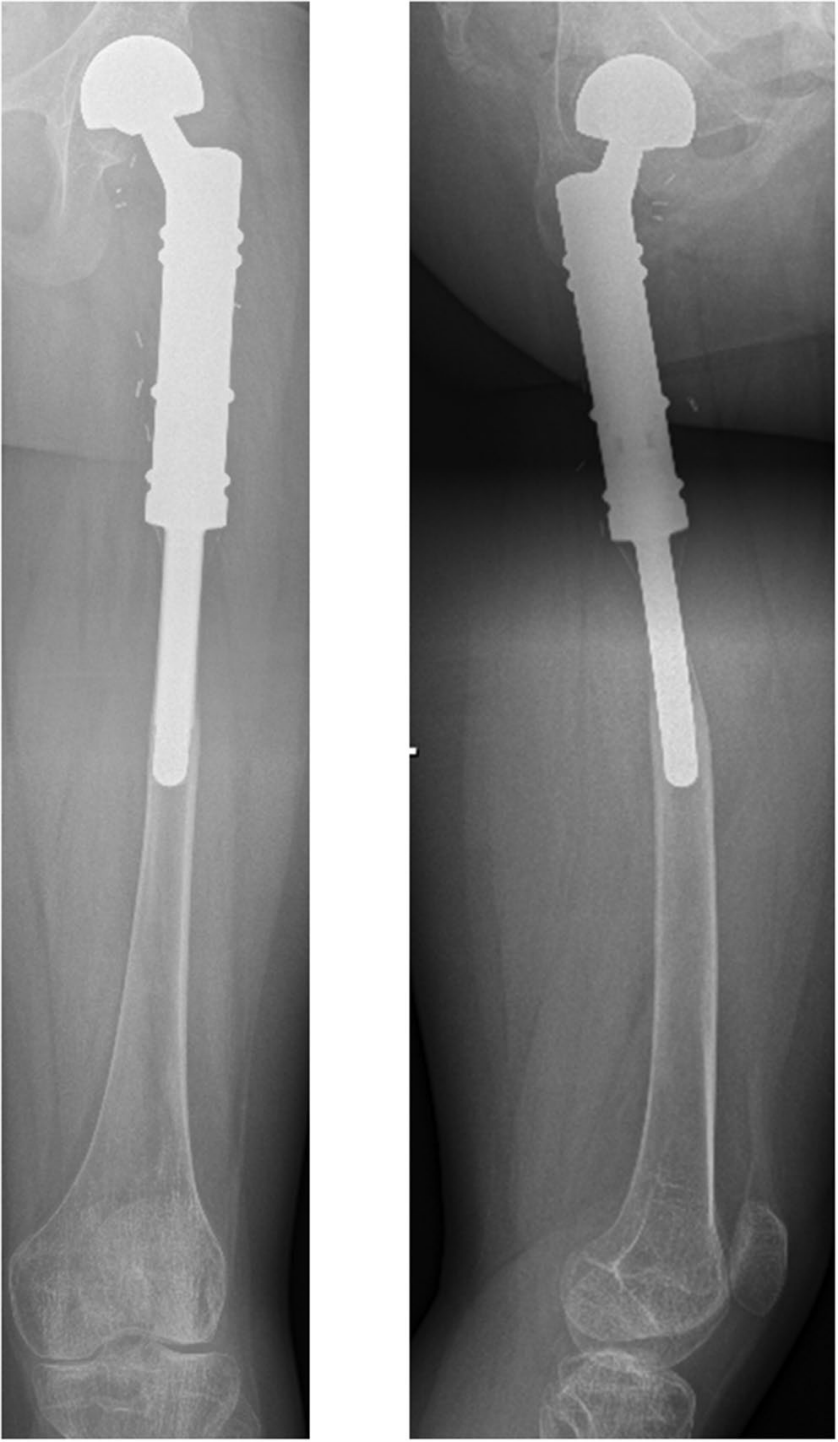
11-year-old patient (#2) with a Ewing’s sarcoma of the left proximal femur. A.p. and lateral x-rays 52 months after the operation (patient age 16 years) with presentation of significant stress shielding in both planes

Stress shielding describes the asymptomatic degeneration of cortical bone adjacent to cementless prosthetic implant stems in pediatric populations. It was observed in ten patients of this cohort (*n* = 10/14; 71.4%) (Fig. 1). Only patients #5 and 9 (reconstructed using hollow custom-made short stems) and patients #11 and 12 (aged 16 and 17 years) were not affected by stress shielding.

### Incidence, management and outcome of secondary deformities

#### Secondary hip dysplasia

**Fig. 2:**
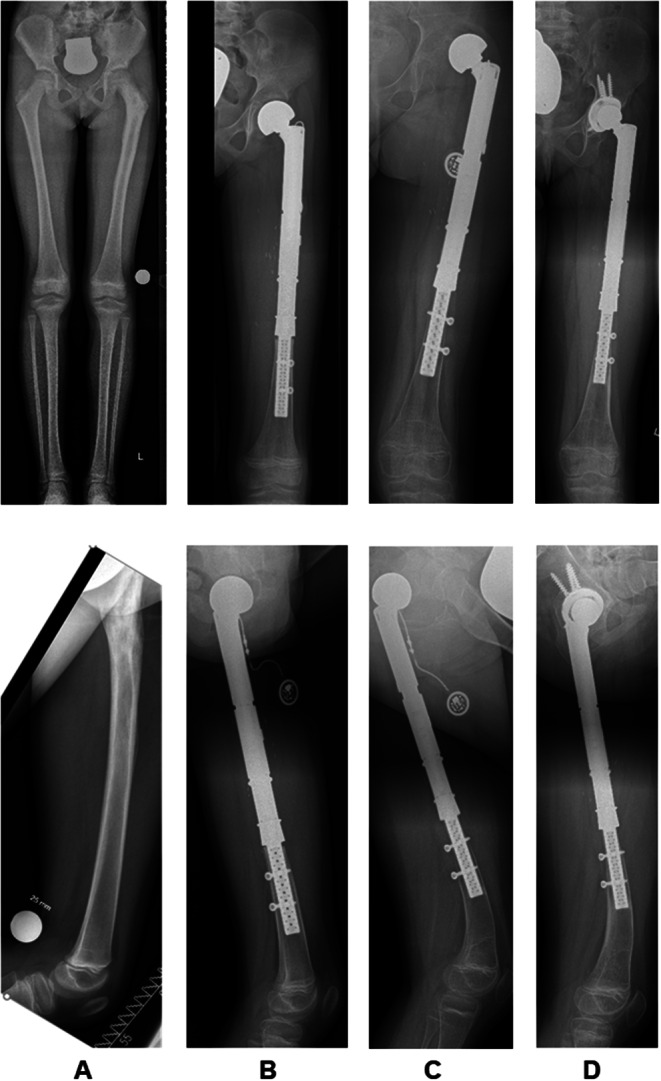
8-year-old patient (#1) with a Ewing’s sarcoma of the left proximal femur. A.p. and lateral x-rays prior to **(A)**, 3 **(B)**, 26 **(C)** and 33 **(D)** months after the 1st and 11 months **(D)** after the 2nd operation. Presentation of secondary hip dysplasia, lateralization and dislocation of the bipolar head in C. Presentation of stress shielding in C and D. Development of a 13° procurvatum flexion deformity in the lateral plane **(C)** and spontaneous correction following the 2nd operation **(D)**

**Fig. 3:**
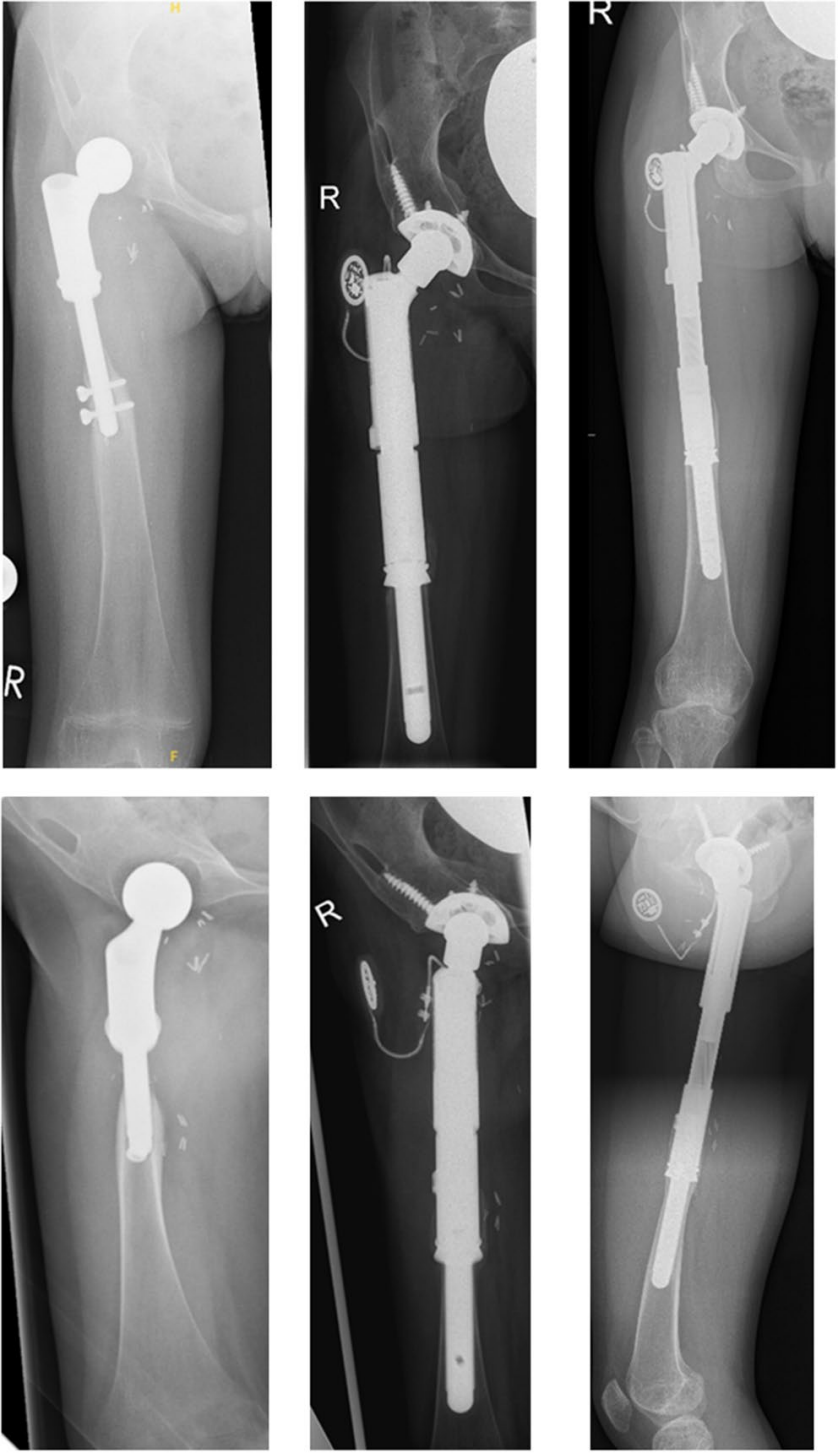
5-year-old patient (#3) with a Ewing’s sarcoma of the right proximal femur. Left: A.p. and lateral x-rays 76 months after the primary operation, 45 months after triple pelvic osteotomy for secondary hip dysplasia and 10 months after attempted reposition in recurrent hip dysplasia. The patient presents with recurrent/persistent hip dysplasia, severe stress shielding and an 8–9 cm limb length discrepancy. Center: A.p. and lateral x-rays 1.5 months after acetabular socket replacement and reconstruction using an expandable megaendoprosthesis. Right: A.p. and lateral x-rays 43 months after acetabular socket replacement and complete lengthening of the prosthesis (5 cm). The growth plates of the lower limbs are closed and both legs of equal length

Secondary hip dysplasia after proximal femur replacement was observed in seven patients (*n* = 7/14; 50%; intraarticular *n* = 7/12; 58.3%) who were aged < 10 years (range 4–8 years) at the time of primary reconstruction (Fig. 2a*/b and 3*). As a result, four of these patients (*n* = 4/7; 57.1%) developed secondary hip dislocations that needed to undergo revision operations after a mean of 26 months (range 16–30 months) after the primary operation (see also Table 1). Soft tissue reconstruction in these cases was performed with or without an attachment tube in three and four cases, respectively. While two patients were reconstructed by endoprosthetic replacement of the dysplastic acetabular socket (#1 and 8), two other patients underwent triple pelvic osteotomies to address hip dysplasia and dislocation at first (#3 and 6). Persistent or rebound hip dysplasia occurred in both cases. Attempted open or closed reductions and acetabular deepening using a reamer did not succeed in both patients and ultimately led to endoprosthetic acetabular socket replacements 24 and 49 months after triple pelvic osteotomy (Fig. 3). Of the other three patients who developed secondary hip dysplasia, two patients did not progress to hip dislocation (#7 and 9). However, patient #9 died of disease 48 months after the primary reconstruction. Patient #10 developed secondary hip dislocation but has not undergone revision surgery so far (see also Table 2). This patient is suffering from delayed physical growth and complex bilateral limb deformities following a delayed fine and gross motor development after the completion of chemotherapy.

**Table 2:** Management of hip dysplasia and dislocation

#	Age (yr)	1st Revision	Time to 1st rev. (mo)	2nd Revision	Time 1st to 2nd rev. (mo)	3rd Revision	Time 2nd to 3rd rev. (mo)	4th Revision	Time 3rd to 4th rev. (mo)
1	8	acetabular socket replacement	30	-	-	-	-	-	-
3	5	triple pelvic osteotomy*	30*	attempted OR in recurrent hip dysplasia*	11*	acetabular socket replacement	13	-	-
6	8	triple pelvic osteotomy*	16*	attempted CR in persistent residual hip dysplasia*	2*	acetabular deepening*	1*	acetabular socket replacement	46
7	7	-	-	-	-	-	-	-	-
8	5	CR, PLPC	1	acetabular socket replacement	27	CR, PLPC	0	-	-
9	4	-	-	-	-	-	-	-	-
10	4	-	-	-	-	-	-	-	-
13	10	CR, ARS	1	OR, ARS	13	-	-	-	-
14	10	CR, PLPC	5	-	-	-	-	-	-

*Abbreviations* #: number; (yr): years; CR: closed reduction; PLPC: pelvis leg plaster cast; ARS: anti-rotation splint; rev.: revision; (mo): months; OR: open reduction. *-revisions performed at a different tertiary center

### Secondary genu valgum

**Fig. 4:**
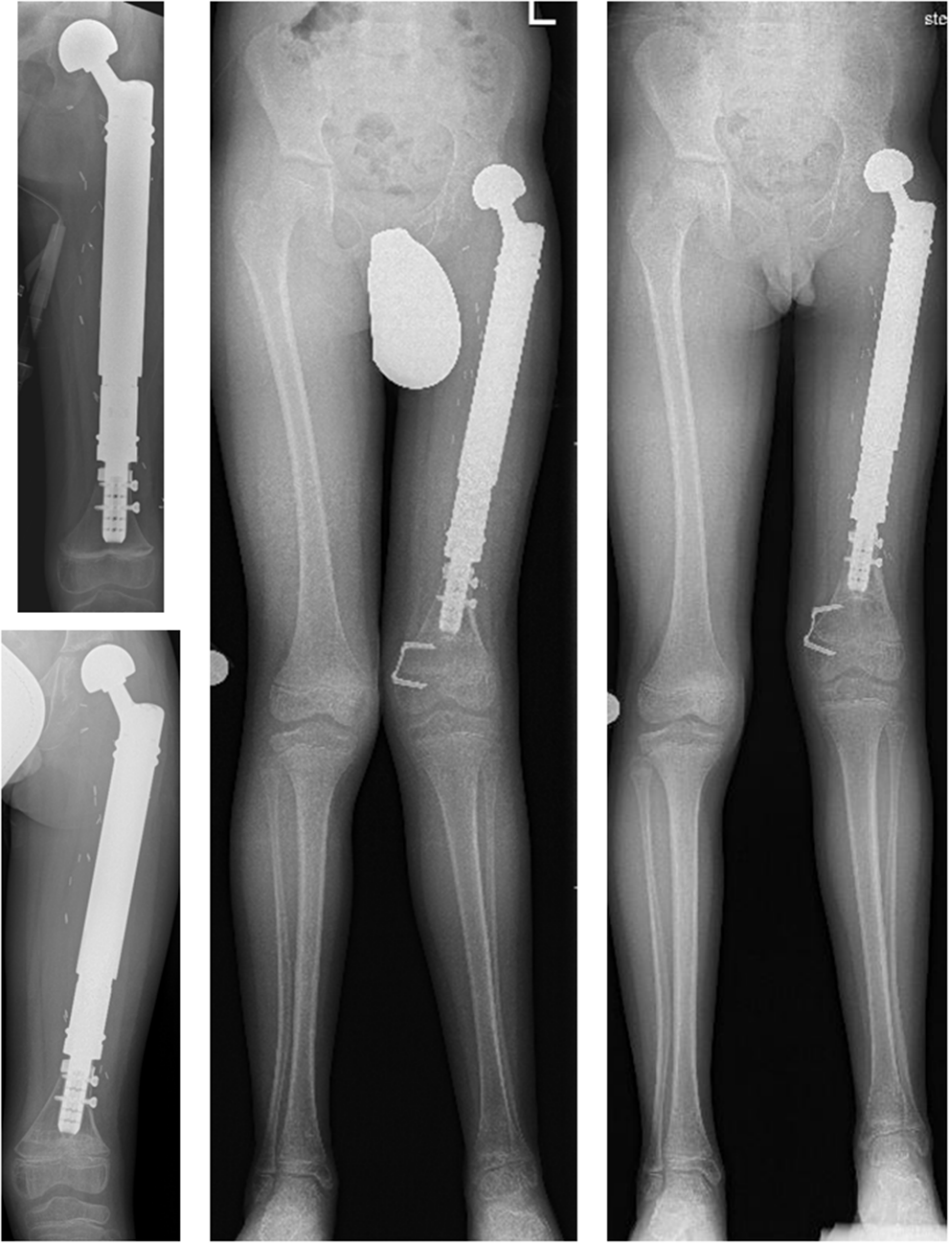
7-year-old patient (#7) with a Ewing’s sarcoma of the left femur. Top and bottom left: A.p. x-rays immediately and 16 months after the operation. A custom-made non-plated hollow stem was used to preserve the distal femoral growth plate. The patient presents with a lateralization of the bipolar head (bottom). Continuous growth of the distal femoral growth plates is apparent (visible temporary growth arrest lines and lengthening of the remaining distal femur). Center: Full-length standing a.p. x-ray 28 months after primary proximal femur replacement and 6 months after temporary hemiepiphyseodesis of the medial distal femoral growth plate for femoral genu valgum. Progressive lateralization of the bipolar head. Right: Full-length standing a.p. x-ray 43 months after primary and 21 months after temporary hemiepiphyseodesis surgery with a visible correction of the genu valgum deformity

Five patients (*n* = 5/12; 41.6%) aged 4–11 years at the time of primary reconstruction developed varying clinical degrees of genu valgum (Table 3). In four patients, a distal femoral cause (pathological mechanical lateral distal femur angle (mLDFA)) was identified. In patient #10 the deformity was bilateral and complex; caused by both pathological mechanical femur and tibia angles. In patient #7 a temporary hemiepiphyseodesis of the distal medial femur epiphysis was performed (Fig. 4), whereas patient #10 underwent bilateral temporary hemiepiphyseodesis of the proximal medial tibia epiphyses despite his young age.

**Fig. 5:**
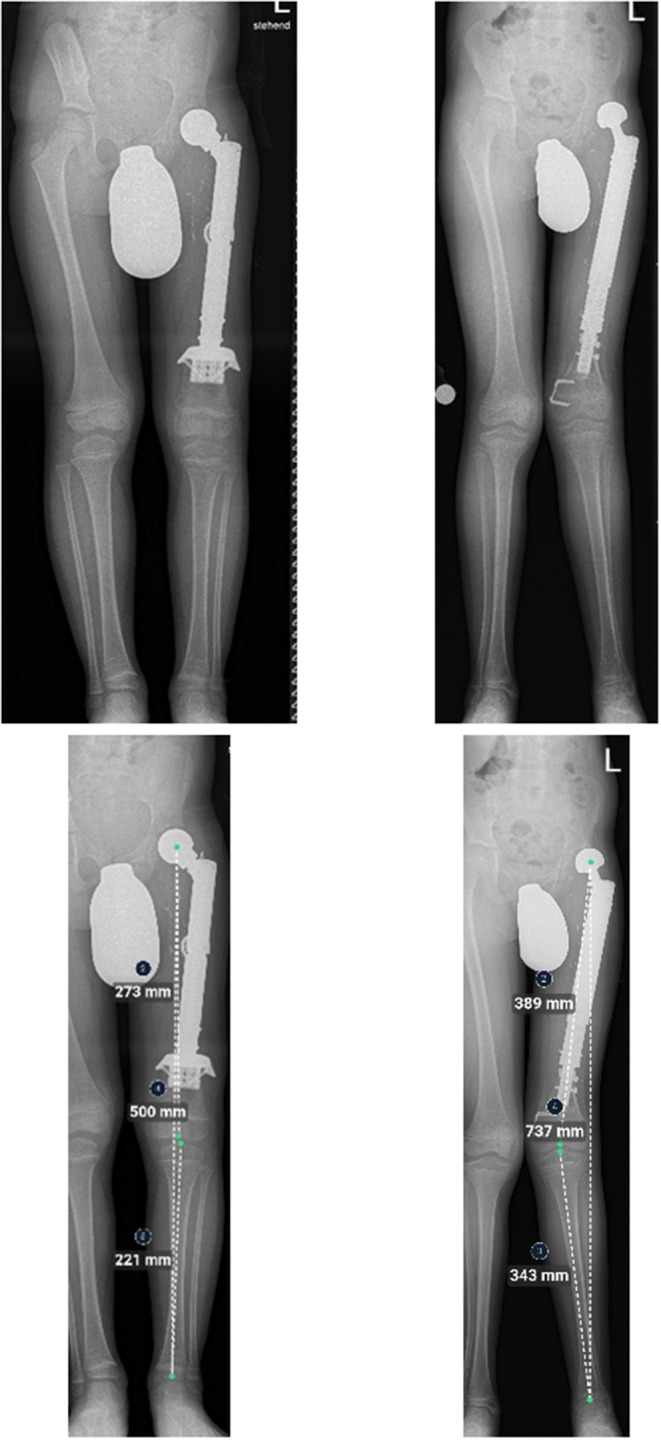
Full-length standing a.p. x-rays of patient #9 (left) and #7 (right). Left: Normal alignment in the frontal plane after proximal femur replacement. The mechanical axis passes through the center of the knee joint. Right: Genu valgum in the frontal plane. The mechanical axis is deviated and passes lateral to the center of the knee joint without making contact with the lateral femur condyle

While pathological mechanical lateral distal femur angles coincided with hip dysplasia in three patients (*n* = 3/7; 42.9%) (Fig. 5), two patients without signs of hip dysplasia were affected by genu valgum as well.

In one patient (#1), who ambulated with a progressing hip dysplasia and slowly progressing hip dislocation, a procurvatum flexion deformity of 13° in the sagittal plane was observed (posterior distal femoral angle (PDFA): 70°; average normal value (range): 83° (79°-87°) (Fig. 2a*/b*) (See Table 3).

**Table 3:** Genu valgum following secondary hip dysplasia

#	Genu valgum	mLDFA (range 85–90°)	1st Revision	Time to 1st rev. (mo)
2	femoral	81.9		
3	femoral	85.4		
5	femoral	80.0		
7	femoral	80.4	temporary hemiepiphyseodesis	22
10	complex	82.0	temporary hemiepiphyseodesis	60

*Abbreviations* #: number; mLDFA: mechanical lateral distal femur angle; (mo): months

## Conclusions

Secondary hip dysplasia was the most frequently observed secondary deformity after intraarticular proximal femur replacement (*n* = 7/12; 58.3%) in this study cohort. All patients affected by secondary hip dysplasia were younger than 10 years of age at primary tumor resection and proximal femur replacement. Because of secondary hip dysplasia, 71.4% of patients (*n* = 5) developed secondary hip dislocations. However, hip dislocations also occurred in four patients without an underlying deformity. These dislocations tended to occur early, within the first five months after the primary operation (in three patients). Both patients with extraarticular resection (#13 and 14) and primary endoprosthetic reconstruction of the acetabulum were affected. Patient #8 (contralateral rotation plasty) was affected both early without an underlying condition, as well as after endoprosthetic acetabular socket reconstruction following the development of secondary hip dysplasia.

Other authors have also observed a high rate of hip dislocation after proximal femur replacement in skeletally immature and mature patients. Van Kampen et al. reported that 75% of their implant failures occurred in patients aged < 11 years in their study (*n* = 40; aged 2–15 years). Implant failure was defined as the necessity for revision of the acetabular implant component. Failure of unipolar replacements was observed in their patient cohort, leading to pain and subluxation within a period of 10 years [11]. Belthur et al. published a rate of hip instability of 44% in their study group of nine patients. They also reported that the use of a bipolar cup allowed a normal acetabular development [12]. Puchner et al. report an overall dislocation rate of 13% in 166 patients after proximal femur replacement (aged 6–84 years) [15].

Therefore, hip instability and dislocation are well-known complications occurring after proximal femur replacement in skeletally immature and adult patients. A relative lack of abductor strength and resection of the joint capsule are usually considered the responsible cause [12]. The use of an attachment tube has been introduced as a means of increasing hip joint stability in the literature [16]. In this study, attachment tubes, which were used depending on the amount of remaining joint capsule (*n* = 8/14; 51.1%), were unable to prevent both hip dislocations and secondary hip dysplasia. In addition, there may be an increased risk of patients having to undergo an open reduction due to attachment tubes posing a repositioning obstacle.

On the other hand, the development of secondary hip dysplasia is unique to skeletally immature patients and especially younger patients (< 10 years) seem to be more at risk. To the best of our knowledge, this deformity associated with pediatric proximal femur replacements has not been reported before. Secondary hip dysplasia and instability in this study occurred both after unipolar and bipolar hemiprosthetic reconstructions of the proximal femur, contrary to what Belthur et al. reported in 2003 [12]. The first patients who presented with secondary hip dysplasia in this cohort underwent triple pelvic osteotomies performed by pediatric orthopedic surgeons (patients #3 and 6; aged 8 and 9 years at the time of repeat surgery). However, the biological growth potential of the acetabular growth plate was underestimated as both patients presented with rebound hip dysplasia in the course of their follow-up. Therefore, triple pelvic osteotomy should probably not be the primary solution considered to address secondary hip dysplasia in patients < 10 years. Attempted open or closed reductions and acetabular deepening using a reamer, performed at another tertiary center, did not succeed in addressing rebound hip dysplasia and do not seem feasible treatment options at all. Ultimately, patients who presented with primary or rebound hip dysplasia underwent acetabular socket replacement at this institution. Unlike patients reported by van Kampen et al., patients in this study did not present with failures of their cementless endoprosthetic acetabular socket reconstruction during follow-up so far.

Based on these experiences, our current standard of care is as follows: skeletally immature patients, who undergo intraarticular resections, are reconstructed using bipolar hemiprosthetic proximal femur replacements to allow an ongoing, albeit dysplastic development of the acetabulum. In the event of secondary hip dysplasia and hip dislocation in patients aged < 10 years, we prefer reconstruction by performing a cementless endoprosthetic acetabular socket replacement. In patients with less remaining growth potential of the acetabulum (> 10 years), we would still consider a triple pelvic osteotomy to postpone endoprosthetic reconstruction as long as possible. To address hip dislocations without an underlying deformity, we have started a more conservative regimen of postoperative rehabilitation including a restriction of the hip joint’s range of motion (as stated in Material & Methods) for twelve rather than six weeks after the operation. So far, a more conservative approach was able to ultimately achieve a stable hip joint despite challenges in compliance of skeletally immature patients.

Another significant secondary deformity following proximal femur replacement in skeletally immature patients observed in this study was the development of secondary genu valgum (41.6%). To our knowledge, an association of proximal femur replacement and genu valgum has neither been observed nor reported before. So far, we hypothesize that a lateralized position of the femoral head (which occurs in hip dysplasia and ultimately hip dislocation) leads to a decentralized increase of load on the lateral aspect of the ipsilateral distal femoral and proximal tibial growth plates. Subsequently, this leads to asymmetrical growth of the affected growth plates (medial > lateral) and development of a genu valgum deformity (see also Fig. 5). Due to a small number of cases, the incidence of this phenomenon needs to be observed and confirmed in the future. In addition, possibly affected patients need to be monitored for contralateral genu valgum, which might be indicative of some cases of genu valgum occurring spontaneously without being associated with or only aggravated by a proximal femur replacement.

The rate of stress shielding observed in this patient cohort was 71.4% and affected patients whose growth plates were open at the time of primary reconstruction (< 16 years). Only two patients, who were reconstructed using shorter-than usual, custom-made hollow stems in metaphyseal stem sites were not affected. While stress shielding has been around for a long time and is often asymptomatic, it likely increases the risk of mechanical failure [17, 18]. In this study, a case of periprosthetic fracture was observed in one patient who suffered from a low-impact fall. To minimize the risk of bone loss associated with stress shielding, further investigations of alternate and site-specific stem designs [18] in pediatric populations seem warranted to reduce stress shielding.

In summary, this study reports two significant secondary deformities (hip dysplasia and genu valgum) following proximal femur replacement in skeletally immature patients that have not been reported before. Over the last few decades, the development of growing, expandable prostheses has led to an increasing number of skeletally immature patients who undergo this type of reconstruction. Due to poor functional outcomes and significant leg length discrepancies, megaendoprosthetic reconstructions were reserved for adult patients in the past, thus leading to very limited experience of complications and secondary deformities arising when implanted in a growing skeleton. Knowledge of specifically pediatric complications such as secondary hip dysplasia, genu valgum, other angular deformities and stress shielding need to be considered in both patient counseling, primary treatment and complication management. Especially patients aged younger than 10 years at the time of primary reconstruction seem to be at risk of developing growth related complications and deformities. The standard of care presented in this study is provisional and will likely undergo further changes as we gain more experience in the endoprosthetic treatment of skeletally immature patients.

### Electronic supplementary material

Below is the link to the electronic supplementary material.

Supplementary Material 1

## Data Availability

The datasets used and analyzed during the current study are available from the corresponding author on reasonable request.
